# Toxicological effects of NCKU-21, a phenanthrene derivative, on cell growth and migration of A549 and CL1-5 human lung adenocarcinoma cells

**DOI:** 10.1371/journal.pone.0185021

**Published:** 2017-09-25

**Authors:** Hsien-Feng Liao, Chun-Hsu Pan, Pei-Yu Chou, Yi-Fong Chen, Tian-Shung Wu, Ming-Jyh Sheu, Chieh-Hsi Wu

**Affiliations:** 1 Cancer Biology and Drug Discovery, China Medical University and Academia Sinica, Taichung, Taiwan; 2 Department of Pharmacy, Yuanli Lee’s General Hospital, Lee’s Medical Corporation, Miaoli, Taiwan; 3 School of Pharmacy, Taipei Medical University, Taipei, Taiwan; 4 Department of Nursing, Hung Kuang University, Taichung, Taiwan; 5 Sports Recreation and Health Management Continuing Studies—Bachelor's Degree Completion Program, Tung Hai University, Taichung, Taiwan; 6 Department of Chemistry, National Cheng Kung University, Tainan, Taiwan; 7 Department of Pharmacy, National Cheng Kung University, Tainan, Taiwan; 8 School of Pharmacy, China Medical University, Taichung, Taiwan; University of South Alabama Mitchell Cancer Institute, UNITED STATES

## Abstract

**Background:**

Chemotherapy insensitivity continues to pose significant challenges for treating non-small cell lung cancer (NSCLC). The purposes of this study were to investigate whether 3,6-dimethoxy-1,4,5,8-phenanthrenetetraone (NCKU-21) has potential activity to induce effective toxicological effects in different ethnic NSCLC cell lines, A549 and CL1-5 cells, and to examine its anticancer mechanisms.

**Methods:**

Mitochondrial metabolic activity and the cell-cycle distribution were analyzed using an MTT assay and flow cytometry in NCKU-21-treated cells. NCKU-21-induced cell apoptosis was verified by Annexin V-FITC/propidium iodide (PI) double-staining and measurement of caspase-3 activity. Western blotting and wound-healing assays were applied to respectively evaluate regulation of signaling pathways and cell migration by NCKU-21. Molecular interactions between target proteins and NCKU-21 were predicted and performed by molecular docking. A colorimetric screening assay kit was used to evaluate potential regulation of matrix metalloproteinase-9 (MMP-9) activity by NCKU-21.

**Results:**

Results indicated that NCKU-21 markedly induced cytotoxic effects that reduced cell viability *via* cell apoptosis in tested NSCLC cells. Activation of AMP-activated protein kinase (AMPK) and p53 protein expression also increased in both NSCLC cell lines stimulated with NCKU-21. However, repression of PI3K-AKT activation by NCKU-21 was found in CL1-5 cells but not in A549 cells. In addition, increases in phosphatidylserine externalization and caspase-3 activity also confirmed the apoptotic effect of NCKU-21 in both NSCLC cell lines. Moreover, cell migration and translational levels of the gelatinases, MMP-2 and MMP-9, were obviously reduced in both NSCLC cell lines after incubation with NCKU-21. Experimental data obtained from molecular docking suggested that NCKU-21 can bind to the catalytic pocket of MMP-9. However, the *in vitro* enzyme activity assay indicated that NCKU-21 has the potential to increase MMP-9 activity.

**Conclusions:**

Our results suggest that NCKU-21 can effectively reduce cell migration and induce apoptosis in A549 and CL1-5 cells, the toxicological effects of which may be partly modulated through PI3K-AKT inhibition, AMPK activation, an increase in the p53 protein, and gelatinase inhibition.

## Introduction

In addition to cigarette smoking, worsening air quality caused by industrial or traffic air pollution has also become an important risk factor for many respiratory diseases including lung cancer. According to the cancer statistic report (from 2009 to 2013) released in 2016 by the North American Association of Central Cancer Registries (NAACCR), the incidence rate and death rate of lung-related cancers were respectively ranked third and first among cancer types. Similar trends were also reported in European and Asia regions based on the GLOBOCAN 2012 report from the International Agency for Research on Cancer (IARC) of the World Health Organization (WHO). More than 80%~85% of lung cancers are categorized as non-small-cell lung carcinoma (NSCLC), and about 40% of lung cancers are adenocarcinomas, a subtype of NSCLC [1]. In general, NSCLC is usually insensitive to chemotherapy and usually accompanied by a high frequency of tumor metastasis [2]. Therefore, increasing numbers of studies have focused on developing novel chemotherapeutic drugs for treating NSCLC to increase the cure rate following conventional surgery [3].

AMP-activated protein kinase (AMPK) plays an important role in regulating cell cycle progression and apoptosis under various stress situations through activation of the proapoptotic p53 protein [4, 5]. An increase in the p53 protein shuts down multiplication of stressed cells and even causes the programmed death of cells in an attempt to eliminate damage and protect the organism. Therefore, the AMPK-activated p53 protein provides a critical hint regarding how to stop tumor development.

The compound, 3,6-dimethoxy-1,4,5,8-phenanthrenetetraone (NCKU-21), is a newly synthesized compound derived from denbinobin, a bioactive phytochemical isolated from *Dendrobium* and *Ephemerantha* (Orchidaceae). Denbinobin was shown to possess many biological functions, such as anti-inflammation [6], anti-angiogenesis [7], antiviral replication [8], and anticancer effects [9–11]. Denbinobin was also found to induce cell death by activating apoptosis in A549 human lung adenocarcinoma cells [9]. Accordingly, our study attempted to evaluate the suppressive activity and toxicological mechanisms of NCKU-21 on cell growth and migration in two different ethnic NSCLC cell lines: A549 and CL1-5 lung adenocarcinoma cells.

## Materials and methods

### Chemicals and reagents

Anti-AMPK (#2793) and anti-phospho-AMPK (#2535) antibodies were purchased from Cell Signaling Technology (Beverly, MA, USA). Primary antibodies for detecting phosphatidylinositol-3-kinase (PI3K; #06–497), AKT (#07–416), phospho-AKT (#07–310), p53 (#CBL404), matrix metalloproteinase-2 (MMP-2; #AB19015), and MMP-9 (#AB19016) were from Millipore (Bedford, MA, USA). The antibody for recognizing GAPDH (#NB300-221) was obtained from Novus Biologicals (Littleton, CO, USA). Anti-rabbit (#GTX213110-01) and anti-mouse (#ab6728) secondary antibodies conjugated to horseradish peroxidase (HRP) were respectively purchased from GeneTex (Irvine, CA, USA) and Abcam (Cambridge, MA, USA). ARP101 (#A4433) was obtained from ApexBio Technology (Houston, TX, USA). All chemicals and reagents were obtained from Sigma-Aldrich (St. Louis, MO, USA) unless otherwise specified.

### Synthesis and identification of NCKU-21

**Fig 1:**
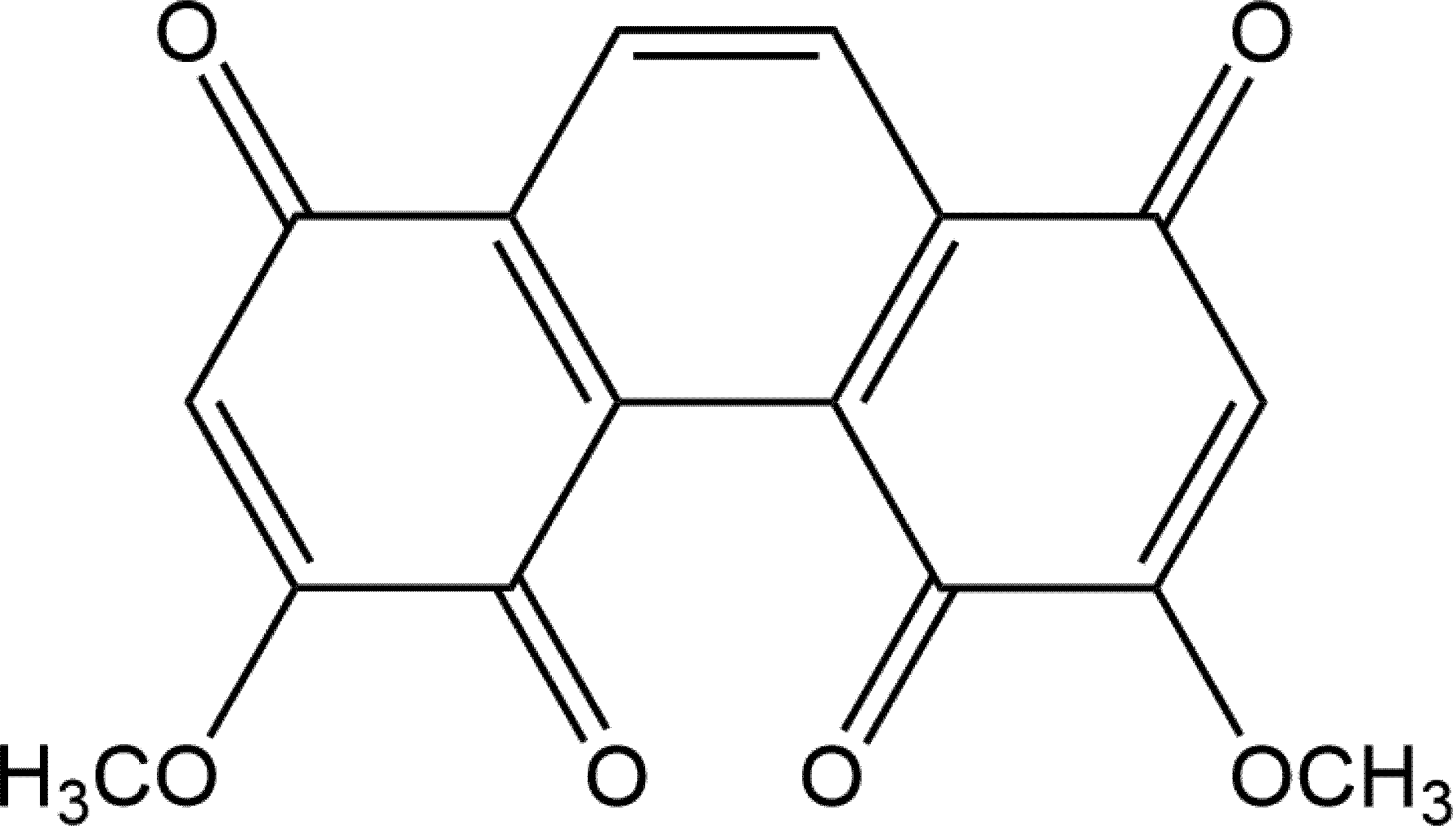
Chemical structure of NCKU-21.

NCKU-21 (Fig 1) was synthesized according to a previous study [12]. Physical and spectroscopic data of NCKU-21 were as follows: mp: 240~242°C; ^1^H-NMR (300 MHz, CDCl_3_) ppm: δ = 3.95 (s, 6 H, OCH_3_), 6.18 (s, 2 H, 2-H, 7-H), 8.39 (s, 2 H, 9-H, 10-H); ^13^C-NMR (75 MHz, CDCl_3_) ppm: δ = 56.81 (OCH_3_), 108.24 (C-2, C-7), 130.44 (C-9, C-10), 133.19 (C-4a, C-4b), 136.61 (C-8a, C-10a), 162.78 (C-3, C-6), 178.81 (C-1, C-8), 182.48 (C-4, C-5); IR (KBr) ν_max_ cm^–1^: 3069, 2935, 2854, 1709, 1690, 1653, 1614, 1455, 1339, 1310, 1252, 1245, 1217, 1179, 1083, 965, 840, 717; ESI-MS: m/z = 299 ([M+H])^+^; HR-ESI-MS: m/z = 299.0556 ([M+H])^+^, calcd. for C_16_H_11_O_6_, 299.0561. The purity used in the following studies was >98% based on a high-performance liquid chromatographic (HPLC) analysis. A stock solution of NCKU-21 was dissolved in dimethyl sulfoxide (DMSO; #D2650), and lower concentrations were further diluted using respective culture medium for the experimental studies.

### Cell culture

A549 cells (#60074) were purchased from the Bioresource Collection and Research Center (BCRC; Hsinchu, Taiwan). CL1-5 cells, an Asian lung adenocarcinoma cell line with highly invasive capacity, were kindly offered by Dr. Chiung-Tong Chen (National Health Research Institutes, Miaoli, Taiwan) [13]. A549 and CL1-5 cells were respectively cultured in F12 medium (#21700–075; Gibco, Grand Island, NY, USA) and RPMI 1640 medium (#318000–22; Gibco). Fetal bovine serum (FBS; US Origin; lot no. 1723586) was purchased from Gibco. All media were supplemented with 10% FBS, 100 U/mL penicillin G, and 10 mg/mL streptomycin. All cells were cultured at 37°C in a humidified incubator with 5% CO_2_, and the culture medium was refreshed every 3 days.

### Measurement of mitochondrial metabolic activity (3-(4,5-dimethylthiazol-2-yl)-2,5- diphenyl-tetrazolium bromide (MTT) assay)

Cells were seeded on 96-well plates (5 × 10^3^ cells/well) after adaptation for 24 hr, and then cells were treated with various concentrations of NCKU-21. At 24 hr after treatment, the culture medium in each well was replaced with 100 μL of diluted MTT (5 mg/mL in phosphate-buffered saline (PBS)). After an additional 3 h in an incubator, the culture medium was removed, and 50 μL of DMSO was added to each well. The absorbance was measured at 570 nm using 650 nm as a reference wavelength. Metabolic activity is presented as a percentage compared to the control group (without NCKU-21 treatment) which was defined as 100%.

### Cell-cycle analysis

Trypsinized cells were washed with PBS and then fixed in 70% ice-cold ethanol overnight. After the ethanol was removed by centrifugation, cell pellets were resuspended in 500 μL of DNA-staining buffer containing 4 μg/mL propidium iodide (PI), 1% Triton X-100, and 0.1 mg/mL RNase. After 30 min of incubation at room temperature in the dark, cells were examined using the FACSCanto flow cytometer system (BD Biosciences, San Jose, CA, USA). The cell-cycle distribution was analyzed using the ModFit LT Program (Verify Software House, Topsham, ME, USA).

### Western blotting

This experiment was carried out as described in our previous study with minor modifications [14]. In brief, trypsinized cells were washed with PBS and then lysed with the PRO-PREP protein extraction solution (#17081; iNtRON Biotechnology, Gyeonggi-Do, South Korea). The cell lysate was centrifuged at 13,000 *×g* and 4°C for 10 min, and then the supernatant was collected. The protein concentration of the supernatant was measured by Bradford’s assay. Aliquots containing 100 μg of protein were separated by 12% sodium dodecylsulfate polyacrylamide gel electrophoresis (SDS-PAGE) gels and electrophoretically transferred to polyvinylidene difluoride (PVDF) membranes (Immobilon-P; Millipore, Bedford, MA, USA). Nonspecific binding sites of the blotted membranes were then blocked with 5% (w/v) nonfat milk (in Tris-buffered saline with 0.1% Tween 20; TBST) at room temperature (RT) for 1 hr. Subsequently, the membrane was incubated with a primary antibody (in TBST) at 4°C overnight and then further incubated with an HRP-conjugated secondary antibody (in TBST) at RT for 1 hr. The membrane was incubated with an Amersham ECL Western Blotting Detection Kit (#RPN2108; GE Healthcare, Chicago, IL, USA), and chemiluminescent signal bands were visualized using the Fujifilm LAS-4000 imaging system (San Leandro, CA, USA). The signal intensity was quantified using Multi Gauge vers. 3.0 analysis software (Fujifilm, San Leandro, CA, USA), and results are presented as the multiple of change compared to that of the control group (without NCKU-21 treatment).

### Annexin V-FITC/PI double-staining

An Annexin V-FITC Apoptosis Detection Kit (#ab14085, Abcam) was used to recognize the translocated phosphatidylserine (PS) of apoptotic cells by a procedure performed according to the manufacturer’s instructions. Briefly, trypsinized cells were re-suspended in 1× binding buffer. After that, 5 μL of Annexin V-FITC and 10 μL of PI (50 μg/mL) were added to each sample. After incubating at RT for 5 min in the dark, the fluorescent intensities of individual samples were measured with a FACSCanto flow cytometer (BD Biosciences, San Jose, CA, USA).

### Measurement of caspase-3 activity

Seeded cells (10^6^ cells/dish) were treated with 2 μM NCKU-21 for 0, 12, and 24 hr. After that, cells were trypsinized and lysed with chilled cell lysis buffer (#1067–100; BioVision, Milpitas, CA, USA). Cell lysates containing 100 μg of protein were diluted to a total volume of 50 μL and then mixed with 50 μL of 2× reaction buffer (#1068–20; BioVision). After that, 5 μL of DEVD-pNA (4 mM in DMSO; #1008; BioVision), a caspase-3 substrate, was added for incubation at 37°C for 2 hr. Caspase activity was determined by measuring the absorbance of the released pNA at 405 nm and is shown as the multiple of change compared to that of the control group (without NCKU-21 treatment).

### Wound-healing assay

Cells were seeded on a 12-well plate (5 × 10^5^ cells/well). After 24 hr of serum-free starvation, a pipette tip was used to create an original cell-free region within the confluent cell monolayer, which was then photographed under a microscope at 40× magnification. After that, cells were treated with various concentrations of NCKU-21. At 18 hr after treatment, the cell number within the original cell-free region was counted on photographic images using the particle analysis function of Image J analytical software (National Institutes of Health (NIH), Bethesda, MD, USA) according to the official web-based manual (https://imagej.net/Particle_Analysis). The degree of cell migration is shown as a percentage of the cell number compared to the control group (without NCKU-21 treatment) which was defined as 100%.

### Molecular docking analysis

A three-dimensional model of human MMP-9 for molecular docking was retrieved from the protein databank (PDB ID: 4XCT), and the image of the predicted interaction was processed using the graphic PYMOL program (Schrödinger, New York, NY, USA). The binding affinity was predicted and calculated using AutoDock Vina, an open-source program for ligand-protein docking and scoring [15]. A value of the root mean squared deviation (RMSD) of < 2.0 Å was considered the best docking pose of candidate ligands, which was very similar to the control ligand.

### MMP-9 activity assay

This experiment was performed using the MMP-9 Colorimetric Drug Discovery Kit (#BML-AK410; Enzo Life Sciences, Farmingdale, NY, USA) according to the manufacturer’s instructions. This experiment was divided into five groups, including blank, control, inhibitor, vehicle, and NCKU-21 groups. Briefly, 90 μl of the reaction solution containing 0.9 mU/μL of recombinant human MMP-9 (#BML-SE360-9090; Enzo Life Sciences) and 1× assay buffer (#BML-KI173-0020; Enzo Life Sciences) in the absence or presence of an inhibitor (2 μM ARP101 in DMSO), vehicle (0.6% DMSO), or NCKU-21 (2 μM in DMSO) was incubated at 37°C for 1 hr. After that, 10 μL of the MMP substrate (#BML-P125-9090; 25 mM) was added, and the absorbance was continually measured at 412 nm (with data recorded every 1 min for 20 min) to obtain the reaction velocity (V) in OD/min. Finally, regulation of MMP-9's enzyme activity by the test compound was calculated using the following equation:

$$MMP\text{-}9\;activity\;\left(\%\right)=V_{test}/V_{control}\times 100\%.$$

### Statistical analysis

Data are presented as the mean ± standard error of the mean (SEM) of at least three independent experiments (*n* = 3/group). Statistical significance was determined by Student's *t*-test, with *P* < 0.05 considered statistically significant.

## Results

### NCI-60 screening of NCKU-21 in NSCLC cells

NCKU-21 was submitted to the National Cancer Institute (NCI, Rockville, MD, USA) of the NIH for anticancer drug screening. A data report (NSC no.: D-752792/1) indicated that NCKU-21 possessed great potential to suppress the cell growth of tested NSCLC cells based on the 50% growth inhibition (GI_50_) value (Table 1). Among the tested cell lines, A549, HOP-62, NCI-H23 and NCI-H522 cells were more susceptible to NCKU-21 treatment according to two cytostatic parameters, including values of the GI_50_ and total growth inhibition (TGI). Of these four cell lines, only HOP-62 and NCI-H522 cells were effectively killed by NCKU-21 based on a cytotoxic parameter, the value of the 50% lethal concentration (LC_50_). However, only A549 cells, a human lung carcinoma cell line from a Caucasian, were easy obtainable among these four cells; Thus, this cell line was selected to explore the anticancer activities and mechanisms of NCKU-21.

**Table 1:** The US National Cancer Institute 60 (NCI-60) drug screening data of NCKU-21 in non-small-cell lung cancer (NSCLC) cells.

Cell line name	Response concentrations (μM)		
	GI_50_	TGI	LC_50_
A549/ATCC	0.426	2.15	100
EKVX	2.89	10.4	36.5
HOP-62	0.308	1.16	4.62
HOP-92	2.98	6.16	21
NCI-H226	1.87	3.66	7.19
NCI-H23	0.24	0.8	>100
NCI-H322M	1.92	4.23	9.33
NCI-H460	1.21	2.95	7.23
NCI-H522	0.329	1.35	5.46

GI_50_, 50% growth inhibition; TGI, total growth inhibition; LC_50_, 50% lethal concentration.

### NCKU-21 inhibits mitochondrial metabolic activity in NSCLC cells

**Fig 2:**
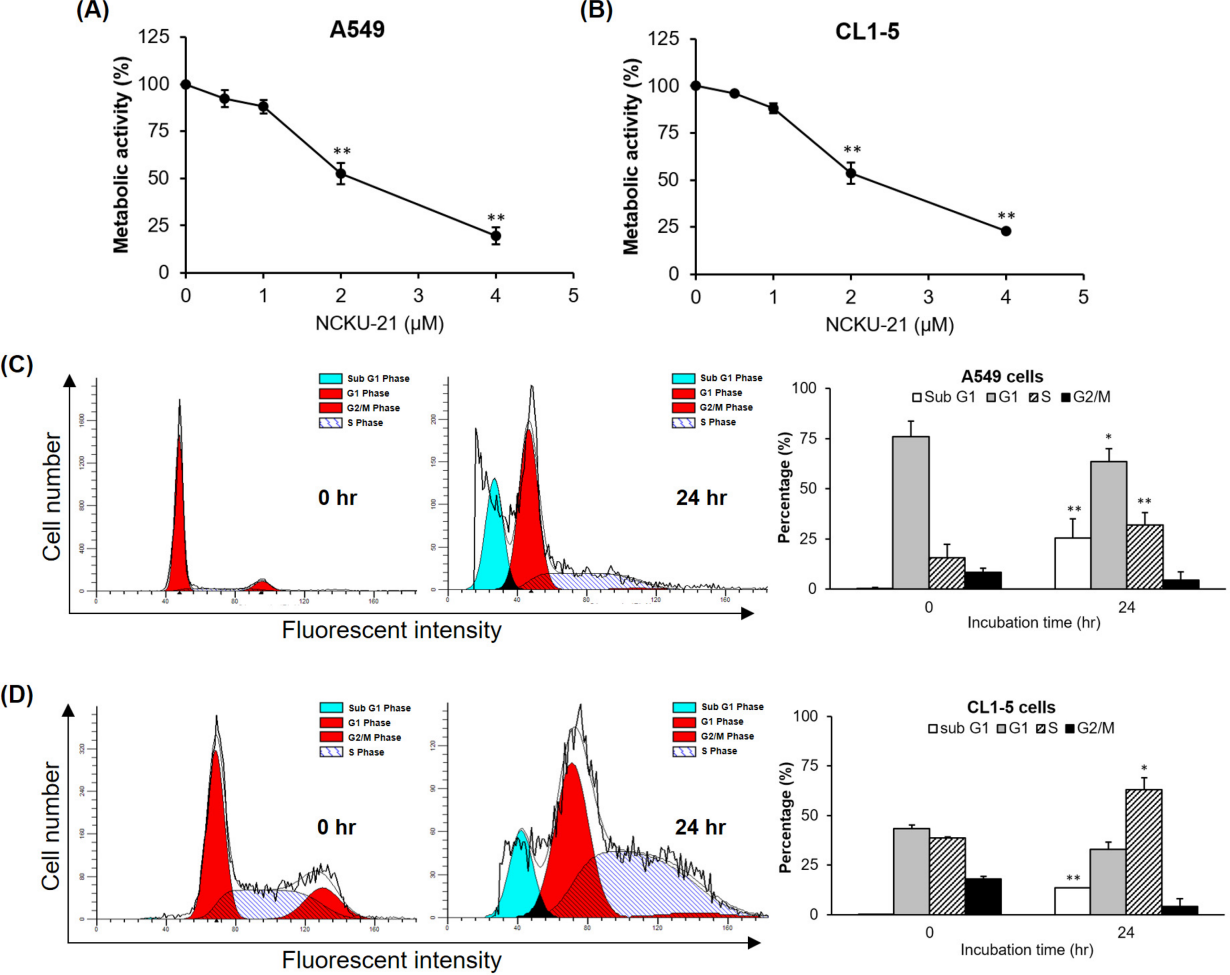
Regulation of the metabolic activity and cell-cycle distribution of NCKU-21. Metabolic activity (A and B) and cell-cycle distribution (C and D) of non-small cell lung cancer (NSCLC) cells (A549 and CL1-5) examined at 24 hr after treatment with NCKU-21. NCKU-21 at a concentration of 2 μM was used to analyze the cell-cycle distribution. * *P* < 0.05 and ** *P* < 0.01, compared to the control group (without NCKU-21 treatment).

Measurement of mitochondrial metabolic activity (by the MTT assay) is widely used as a rough evaluation of cell viability. To evaluate the inhibitory effect of NCKU-21 on metabolic activity, another NSCLC cell line, CL1-5 cells, an Asian lung adenocarcinoma with a highly invasive capacity, were also included. Cells were treated with various concentrations (0.5, 1, 2, and 4 μM) of NCKU-21 for 24 hr, and metabolic activity was analyzed with an MTT assay. Our results showed that the inhibitory effects of NCKU-21 on metabolic activities of two different ethnic NSCLC cell lines (A549 and CL1-5 cells) were very similar (Fig 2A and 2B). At 24 hr after treatment, the half-maximal inhibitory concentration (IC_50_) values of NCKU-21 against A549 and CL1-5 cells were 2.41 and 2.49 μM, respectively.

### NCKU-21 induces apoptosis of A549 and CL1-5 cells

To understand whether NCKU-21-induced growth inhibition against NSCLC cells was due to a cytotoxic or cytostatic effect, we evaluated phase distributions of the cell cycle in NSCLC cells 24 hr after treatment with 2 μM of NCKU-21. Experimental data indicated that the percentage of cells in the sub-G_1_ phase was obviously elevated in both NSCLC cell lines after 24 hr of treatment with NCKU-21 (Fig 2C and 2D), the results of which suggested that hypodiploid cells were elevated after being treated with NCKU-21 for 24 hr in both NSCLC cell lines. Therefore, NCKU-21-induced growth inhibition was mainly mediated by a cytotoxic effect.

### Regulation of growth control-associated proteins by NCKU-21 in A549 and CL1-5 cells

**Fig 3:**
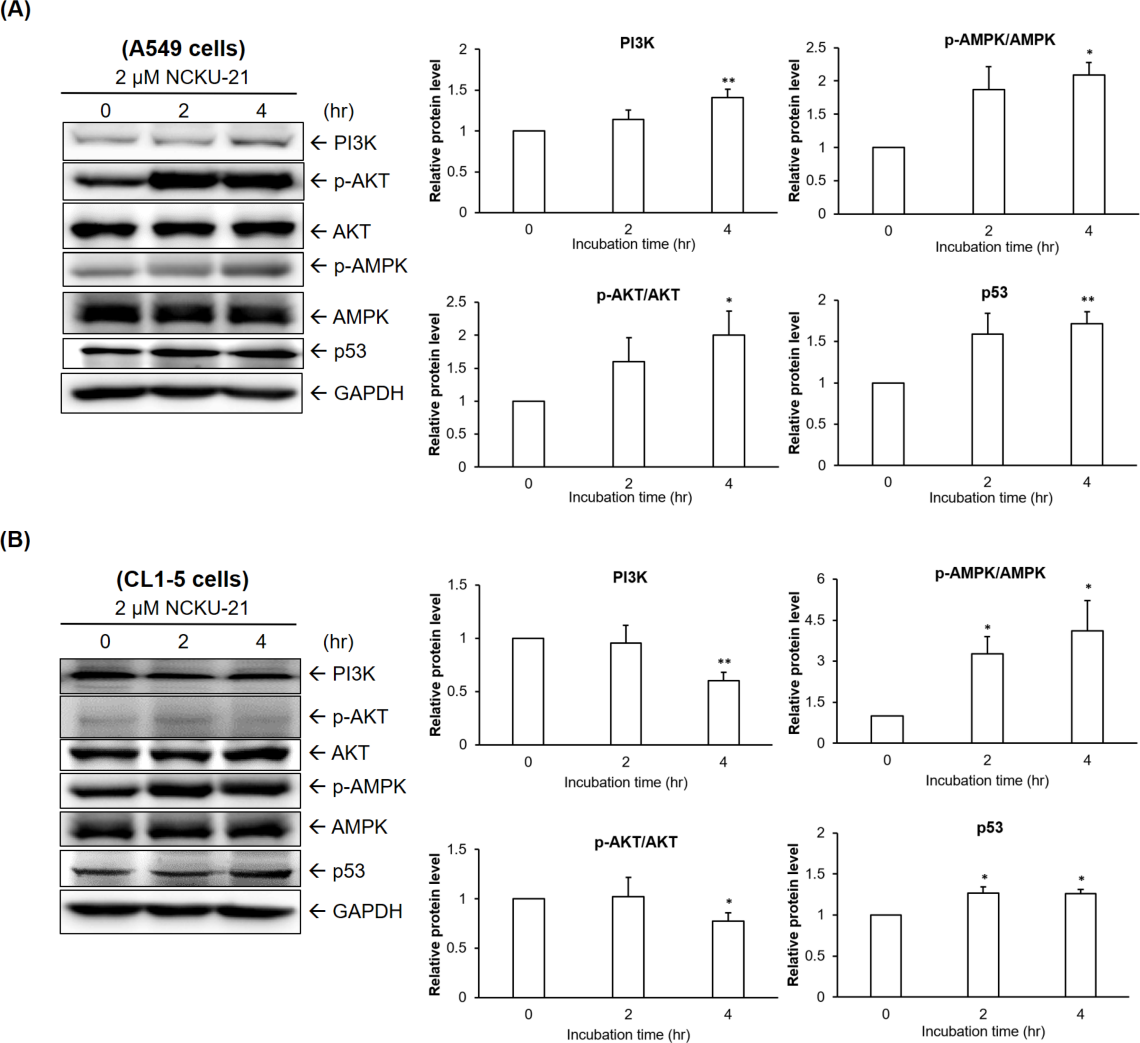
Regulation of growth control-associated proteins by NCKU-21 in A549 and CL1-5 cells. Expressions and activation levels of relative proteins were examined in A549 (A) and CL1-5 (B) cells after treatment with NCKU-21. Total and phosphorylated levels of the tested proteins were examined in cells treated with NCKU-21 for 24 hr and 30 min, respectively. * *P* < 0.05 and ** *P* < 0.01, compared to the control group (without NCKU-21 treatment).

Expression or activation levels of proteins associated with growth regulation were examined in A549 and CL1-5 cells after treatment with NCKU-21. In A549 cells, our study showed that NCKU-21 increased AMPK activation and p53 protein levels, whereas NCKU-21 had no effect on suppressing serum-stimulated PI3K-AKT activation (Fig 3A). In CL1-5 cells, NCKU-21 exhibited the same regulatory patterns on AMPK and p53 and also suppressed the serum-activated PI3K-AKT pathway (Fig 3B).

### NCKU-21 promotes PS externalization and caspase-3 activity in A549 and CL1-5 cells

**Fig 4:**
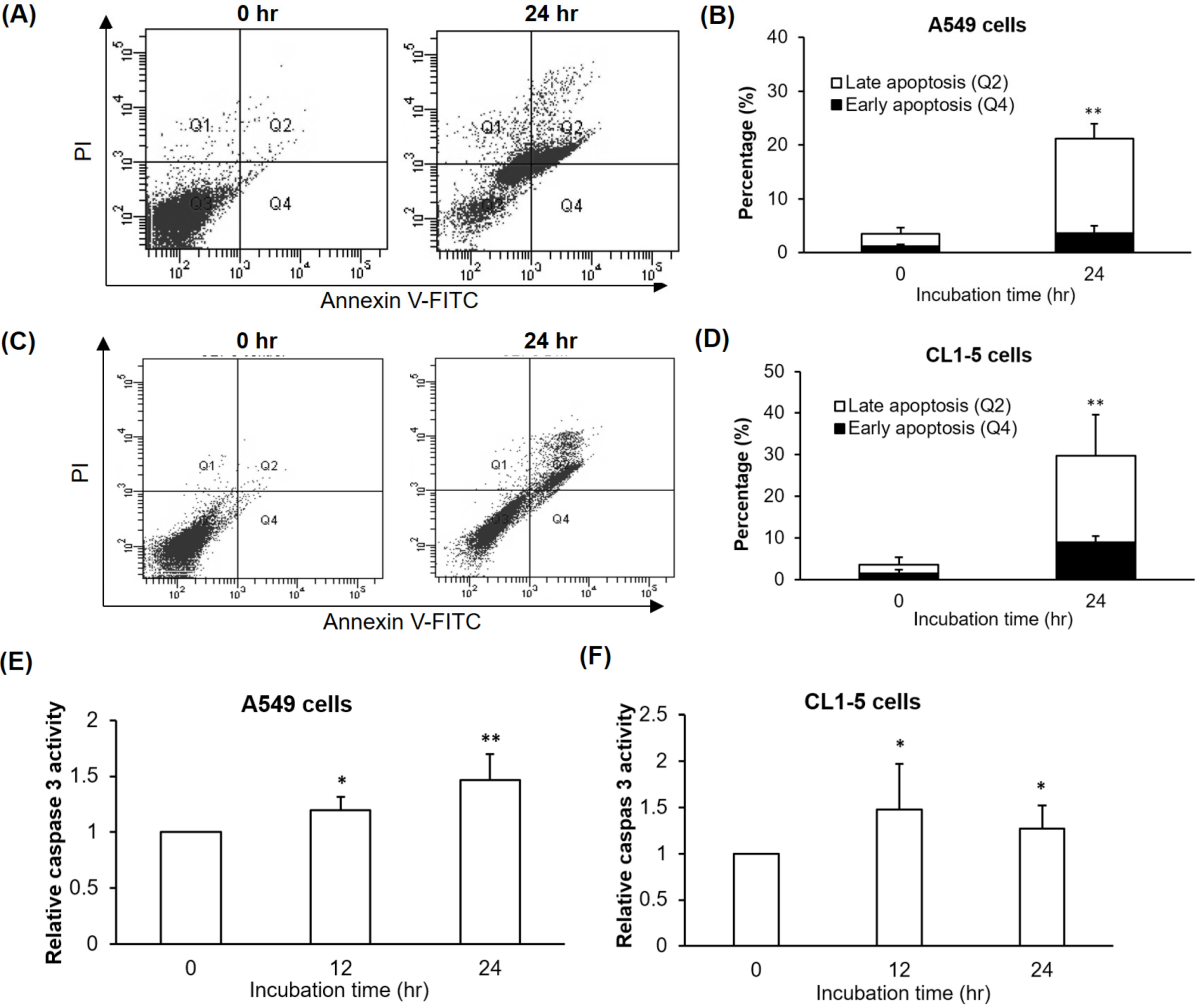
Regulation of phosphatidylserine (PS) externalization and caspase-3 activity by NCKU-21 in A549 and CL1-5 cells. Membrane translocation of PS was determined by staining with Annexin V-FITC/propidium iodide (PI) and then analyzed by flow cytometry in A549 and CL1-5 cells treated with 2 μM of NCKU-21 for 24 hr. Data are displayed as a representative dot plot and statistical histogram in A549 (A and B) and CL1-5 (C and D) cells. Relative caspase-3 activity was measured in A549 (E) and CL1-5 (F) cells treated with 2 μM of NCKU-21 for 24 hr. ** *P* < 0.01, compared to the control group (without NCKU-21 treatment).

To confirm the apoptotic effect of NCKU-21 in the NSCLC cell lines, we evaluated the membrane translocation of PS, an early marker of cell apoptosis, and activity change of caspase-3, a critical executive caspase required for DNA fragmentation, in A549 and CL1-5 cells incubated with 2 μM of NCKU-21. Results obtained from Annexin V-FITC/PI double-staining revealed that percentages of cell populations at the early (quadrant 4) and late (quadrant 2) stages of cell apoptosis increased after NCKU-21 incubation in A549 (Fig 4A and 4B) and CL1-5 cells (Fig 4C and 4D). Similarly, NCKU-21 incubation was also found to stimulate large increases in caspase-3 activity in A549 and CL1-5 cells (Fig 4E and 4F). Together, these results suggest that the NCKU-21-induced cytotoxic effect in A549 and CL1-5 cells might partly be attributed to activation of caspase-dependent programmed cell death.

### NCKU-21 suppresses cell migration and gelatinase expressions in A549 and CL1-5 cells

**Fig 5:**
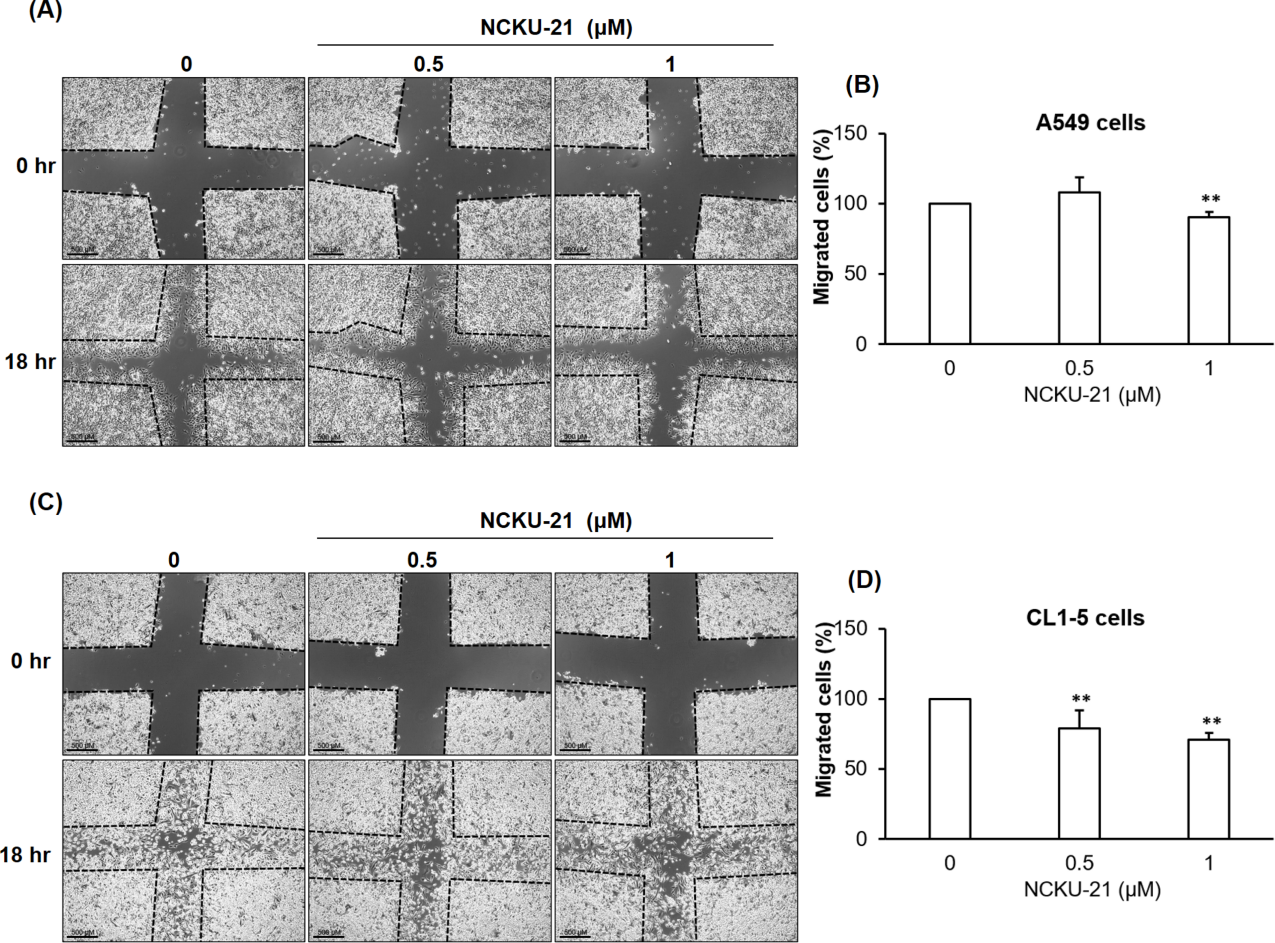
Inhibitory effect of NCKU-21 on cell migration of A549 and CL1-5 cells. Cell migration levels were examined using a wound-healing assay in A549 and CL1-5 cells treated with NCKU-21 for 18 hr. Pictures were acquired from A549 (A) and CL1-5 (C) cells after 0 and 18 hr of NCKU-21 treatment. Statistical results obtained from A549 (B) and CL1-5 (D) cells are presented as histograms. Scale bar: 500 μm. ** *P* < 0.01, compared to that of the group without NCKU-21 treatment.

**Fig 6:**
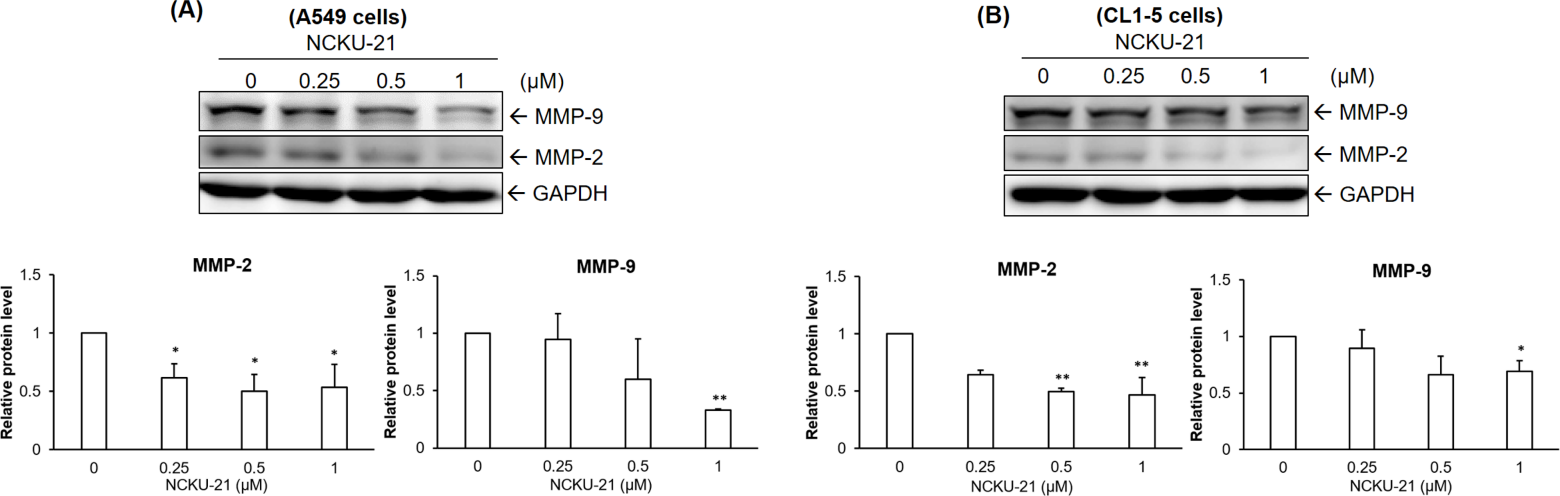
Regulation of cell migration-associated proteins by NCKU-21 in A549 and CL1-5 cells. Protein expressions of matrix metalloproteinase-2 (MMP-2) and MMP-9 were analyzed in A549 (A) and CL1-5 (B) cells treated with NCKU-21 for 24 hr. * *P* < 0.05 and ** *P* < 0.01, compared to the control group (without NCKU-21 treatment).

To investigate the suppressive potential of NCKU-21 against NSCLC migration, two highly aggressive lung cancer cell lines, A549 and CL1-5 cells, were further evaluated to examine whether their cell-migratory ability was affected by treatment with NCKU-21. Results indicated that NCKU-21 at the doses with minimal metabolic inhibition and cytotoxicity significantly decreased serum-stimulated cell migration of NSCLC cells during an 18-hr time course (Fig 5). Moreover, protein expressions of gelatinases (MMP-2 and MMP-9) were obviously inhibited in NSCLC cells incubated with NCKU-21 at concentrations below the IC_50_ value (Fig 6).

### NCKU-21 has great potential to bind with the catalytic domain of MMP-9 according to a molecular docking model

**Fig 7:**
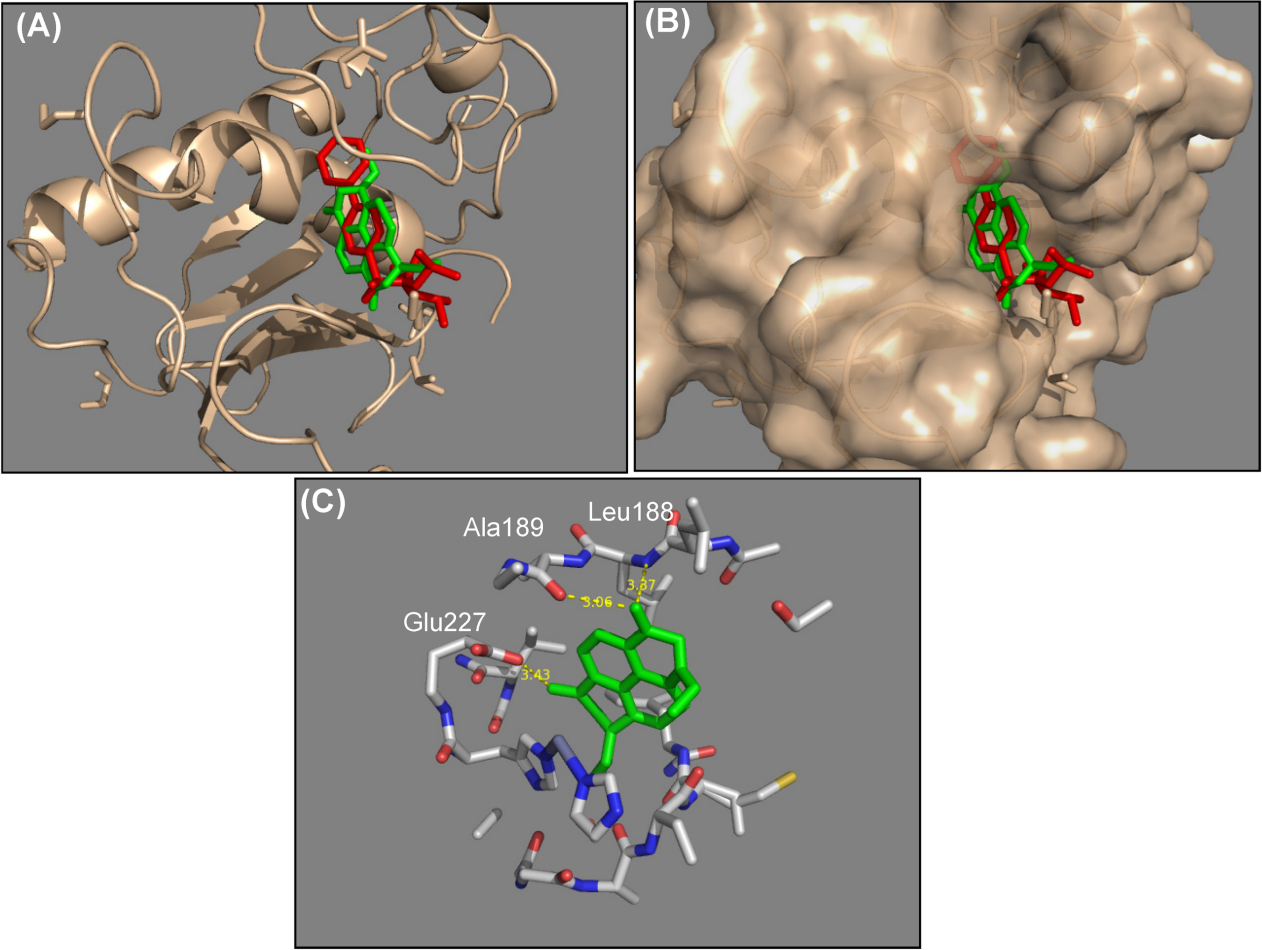
Molecular docking analysis of matrix metalloproteinase-9 (MMP-9) and NCKU-21. Docking poses of ARP101 (red color), a known MMP-9 inhibitor, and NCKU-21 (green color) within the pocket domain of MMP-9 are shown in a stick model (A) and in a surface model (B). Residues that may be involved in the interactions of compound binding are shown in the stick model (C), and hydrogen-bond interactions, oxygen atoms, nitrogen atoms, and hydrogen atoms are respectively indicated with yellow dashed lines, and red, blue, and white colors.

**Fig 8:**
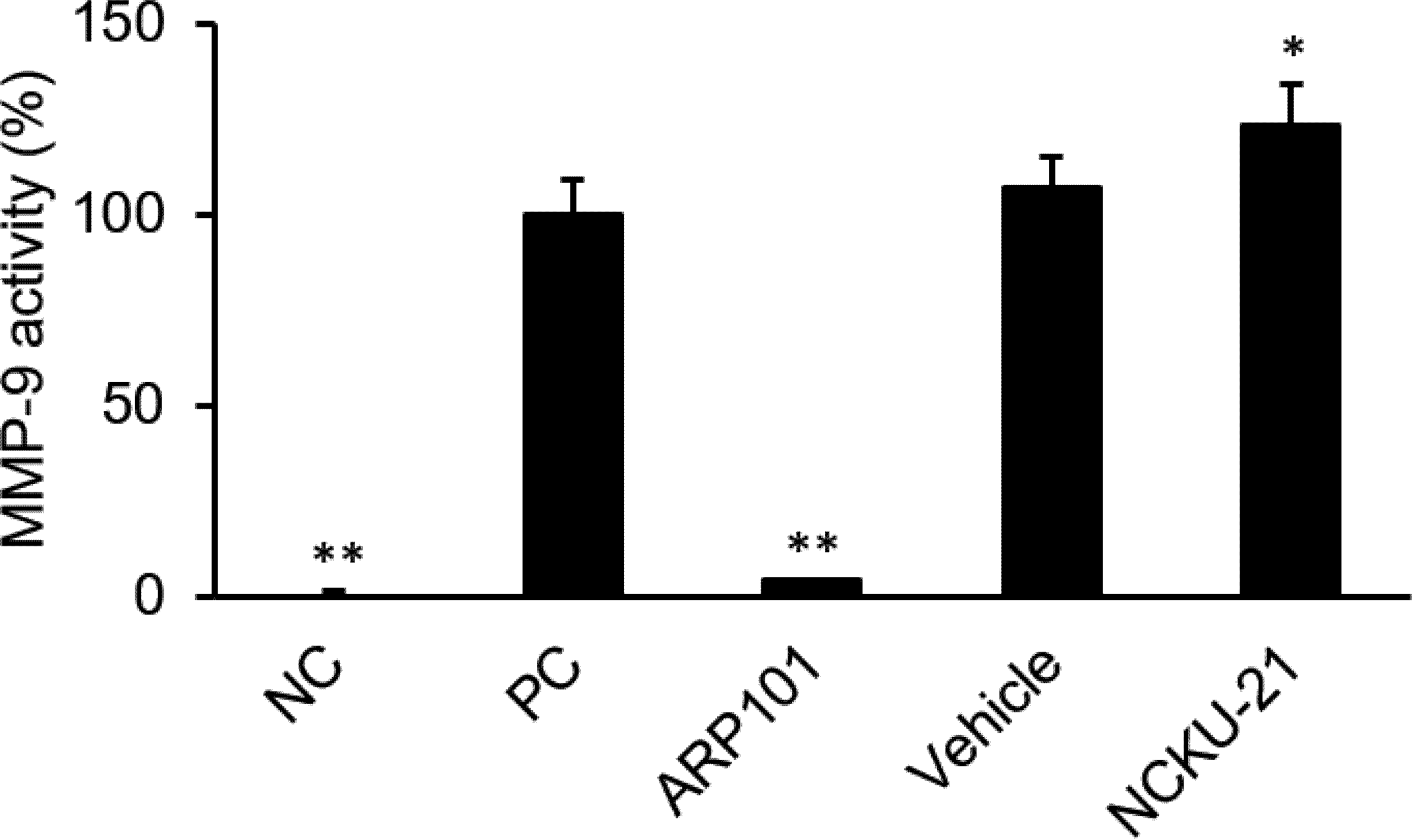
Regulation of human matrix metalloproteinase-9 (MMP-9) activity by NCKU-21. NC (negative control), the group under the original reaction condition without MMP-9 added; PC (positive control), the group under the original reaction condition with no inhibitors or test compounds added; ARP101, PC group treated with 2 μM ARP101; Vehicle, PC group treated with 0.6% DMSO; NCKU-21, PC group treated with 2 μM NCKU-21. ** *P* < 0.01 compared to the control group.

According to data retrieved from the Oncomine data-mining platform (Compendia Bioscience, Ann Arbor, MI, USA), Garber et al. reported that the transcriptional level of human MMP-9 greatly increased in lung cancer tissues (S1 Fig) [16]. Thus, we evaluated whether NCKU-21 has the potential to interact with MMP-9 and then to interfere with MMP-9 activity in NSCLC cells. The binding interaction between NCKU-21 and MMP-9 was evaluated with a computational docking model. As shown in Fig 7, the virtual docking model indicated that NCKU-21 could successfully bind to the catalytic domain of MMP-9, like the original inhibitor, ARP101 [17]. In addition, the interaction prediction indicated that hydrogen bonds would be formed between NCKU-21 (at positions C1 and C8) and MMP-9 (at three positions of Leu188, Ala189, and Glu227 within the catalytic domain). Thus, the electrostatic attraction caused by the hydrogen bonds would effectively enhance the affinity of NCKU-21 to bind to the catalytic pocket. The binding energy of the NCKU-21/MMP-9 complex in the most stable mode was calculated to be -8.3 kcal/mol. Therefore, regulation of MMP-9's activity by NCKU-21 was further evaluated. *In vitro* data indicated that MMP-9 activity could be significantly reduced by about 96% in the group treated with an MMP-9 inhibitor (2 μM ARP101). However, the enzyme activity was enhanced after 2 μM of NCKU-21 treatment (Fig 8).

## Discussion

The hallmark capabilities of cancer, including sustaining proliferative signaling, evading growth suppressors, resisting cell death, enabling replicative immortality, inducing angiogenesis, activating invasion and metastasis, reprogramming energy metabolism, and escaping immune destruction, were proposed to increase tumor survival and progression [18]. It was reported that the effect of single-target chemotherapy is often offset by complementary enhancement of other hallmark capabilities [19, 20]. Accordingly, developing or screening an anticancer compound with multiple regulatory actions, just like combination or cocktail therapy for targeting multiple hallmarks, will be helpful in greatly increasing cancer cure rates. Our study showed that NCKU-21 possesses effective potency against human NSCLC cells with regard to a number of hallmarks, including inducing cell apoptosis and inhibiting cell migration (Figs 2, 4 and 5).

AMPK is a critical energy sensor for maintaining intracellular energy homeostasis. Under some energetic or oxidative stresses, such as starvation and hypoxia, it can be activated to stimulate catabolic responses, e.g., fatty acid oxidation and glycolysis, and inhibit anabolic reactions, e.g., gluconeogenesis and glycogen synthesis. AMPK was reported to possess critical functions of regulating many cellular processes, such as cell-cycle arrest and apoptosis, and has thus become a therapeutic target for treating malignancies [21]. These regulatory mechanisms of AMPK-induced cell-cycle arrest and apoptosis may be mediated by AMPK-induced p53 activation [22, 23]. Activation of the p53 protein, a tumor suppressor and transcription factor, was found to induce expressions of multiple target molecules that promote cell-cycle arrest or apoptosis [24]. In the present study, increases in AMPK activation and p53 protein levels were detected in A549 and CL1-5 cells after incubation with NCKU-21. In this way, AMPK-p53 activation may contribute to NCKU-21-mediated toxicological effects to induce apoptosis (Fig 4), migration inhibition (Fig 5), and gelatinase downregulation (Fig 6).

The PI3K-AKT pathway is also a pivotal intracellular signaling cascade involved in cell proliferation and survival (apoptosis inhibition). Alteration or mutation of the PI3K-AKT signaling pathway was linked to resistance against chemotherapy-induced apoptosis in human cancers [26]. Activated PI3K-AKT identified in NSCLC was related to a poor prognosis of patients [27, 28]. Therefore, specific inhibitors targeting upstream regulators or downstream effectors of the PI3K-AKT signaling pathway, such as human epidermal growth factor receptor 2 (HER2) or mammalian target of rapamycin (mTOR), were developed as novel strategies to increase the chemotherapeutic sensitivity of cancer cells [26, 29]. Moreover, previous studies also reported that the PI3K-AKT pathway can participate in the invasion and metastasis of cancer cells through increasing expression levels of gelatinases (MMP-2 and MMP-9) [30–32]. In our study, NCKU-21 suppressed the PI3K-AKT signaling pathway only in CL1-5 cells (Fig 3B), the regulation of which might partially involve reductions of cell migration and gelatinase expressions (Figs 5 and 6). However, NCKU-21 had no inhibitory effect on serum-induced PI3K-AKT activation in A549 cells (Fig 3A). One possible reason is that A549 NSCLC cells carry the KRAS mutation. KRAS mutations, identified in approximately 30% of human cancers, may be associated with resistance to PI3K/AKT/mTOR inhibitors [33–36]. Similarly, loss of the phosphatase and tensin homolog (PTEN), a tumor-suppressor protein with a suppressive function against PI3K-mediated AKT activation, is also a potential predictive marker of a poor prognosis in NSCLC patients [27]. CL1-5 cells are characterized by a mutation in PTEN [37]. These results imply that suppression of PI3K-AKT activation by NCKU-21 targets KRAS or other upstream regulators rather than the PTEN protein in CL1-5 cells.

MMPs, a family of zinc-dependent extracellular matrix degradation enzymes, were demonstrated to be involved in some cellular physiological processes, such as cell proliferation, migration and invasion [38, 39]. Among these enzymes, abnormal expressions or activations of gelatinases, MMP-2 (gelatinase A) and MMP-9 (gelatinase B), were reported to be associated with numerous disease conditions, such as cancer, cardiovascular diseases and Alzheimer's disease [40–42]. A previous study mentioned that metformin, an AMPK activator, can block melanoma from invasion and metastasis by suppressing the activities and expressions of gelatinases (MMP-2 and MMP-9) in an AMPK-p53-dependent manner [25]. Our study revealed that NCKU-21 can also induce AMPK activation and p53 expression (Fig 3), and these regulatory mechanisms might partially explain the inhibitory effect of NCKU-21 on translational levels of MMP-2 and MMP-9 (Fig 6). In addition, the pathological roles of gelatinases in tumor migration and invasion are well-documented [41]. Thereby, decreasing expressions of gelatinase proteins may participate in the inhibitory effect of NCKU-21 on cell migration in the tested NSCLC cells (Figs 5 and 6). In addition, the experimental results obtained from molecular docking showed that NCKU-21 can interact with the pocket domain of the MMP-9 protein and hence has the potential to interfere with MMP-9’s activity (Fig 7). However, unexpected data from *in vitro* study implied that NCKU-21 might possess a promoting effect to increase MMP-9 activity (Fig 8). This controversial result might have been caused by some experimental limitations. First, the three-dimensional (3D) molecular structure of the docked protein can change with different environmental factors, such as the solvent, resulting in alterations of ligand-protein interactions. Hence, the molecular structure of the MMP-9 protein should differ between the crystallized structure (ID: 4XCT) from the PDB database and the experimental measurement of *in vitro* MMP-9 activity in the present study. Second, the MMP-9 protein is activated by cleavage of the native form (inactive status; 92 kDa). In our study, the MMP-9 protein supplied within the kit is a recombinant truncated protein (catalytic domain alone; 39 kDa). Thus, its molecular conformation might differ from the original active MMP-9 protein, which might have the chance to induce MMP-9 activity by creating a new interaction (allosteric regulation) between the ligand (NCKU-21) and protein.

In contrast to normal cells, cancer cells are characterized by persistently elevated levels of oxidative stress due to higher intracellular metabolism, and thus, they may be relatively more sensitive than normal cells to an increase in oxidative stress or a redox imbalance [43, 44]. Thus, disturbing ROS homeostasis has become one anticancer strategy for developing novel chemotherapy agents [45]. Oxidative stress and a hypoxic situation are partially responsible for triggering activation of AMPK or increases in activation of it and the p53 protein [46]. Our supplementary data suggested that NCKU-21 stimulation markedly induced accumulation of ROS within A549 and CL1-5 cells after 30 min of incubation (S2 Fig), the effect of which may partially explain why AMPK and p53 were upregulated in A549 and CL1-5 cells after NCKU-21 treatment (Fig 3).

## Conclusions

NCKU-21 induced apoptosis and inhibited cell migration in the A549 and CL1-5 human lung adenocarcinoma cell lines, the toxicological activities of which may be partially mediated through suppression of PI3K-AKT, increases in AMPK phosphorylation and the p53 protein, and inhibition of gelatinases. Our findings suggest that low-dose NCKU-21 possessed anticancer activity and great potential for use as a chemotherapeutic agent to treat different NSCLC cells.

## Supporting information

S1 Fig Transcriptional expression of human matrix metalloproteinase-9 (MMP-9) in lung cancers. This was a study by Garber et al [16]. Statistical data were retrieved and analyzed from Oncomine, a cancer microarray database and integrated data-mining platform, and results are presented as a box plot diagram. Lung tissues obtained from normal and four lung tumor types were included. 0, normal (n = 6); 1, large-cell lung carcinoma (n = 4); 2, lung adenocarcinoma (n = 42); 3, small cell lung carcinoma (n = 5); 4, squamous cell lung carcinoma (n = 16). The log2 medium-centered intensity means the intensities were processed by median centering (normalization) and then log2-based transformation.
(TIF)

S2 Fig Inductive effect of NCKU-21 on reactive oxygen species (ROS) in A549 and CL1-5 cells. ROS production and changes were measured by flow cytometry in cells treated with 2 μM of NCKU-21 for the indicated period (0~4 h). A detailed description of the measurement of the ROS level is provided in “Supplementary information”. * *P* < 0.05 and ** *P* < 0.01, compared to the control group (without NCKU-21 treatment).
(TIF)

S1 File (PDF)
