# Supplementary figures and images for: Toxicological effects of NCKU-21, a phenanthrene derivative, on cell growth and migration of A549 and CL1-5 human lung adenocarcinoma cells
Source: PLoS One. 2017 Sep 25;12(9):e0185021. doi: 10.1371/journal.pone.0185021 (PMC5612657; doi:10.1371/journal.pone.0185021)

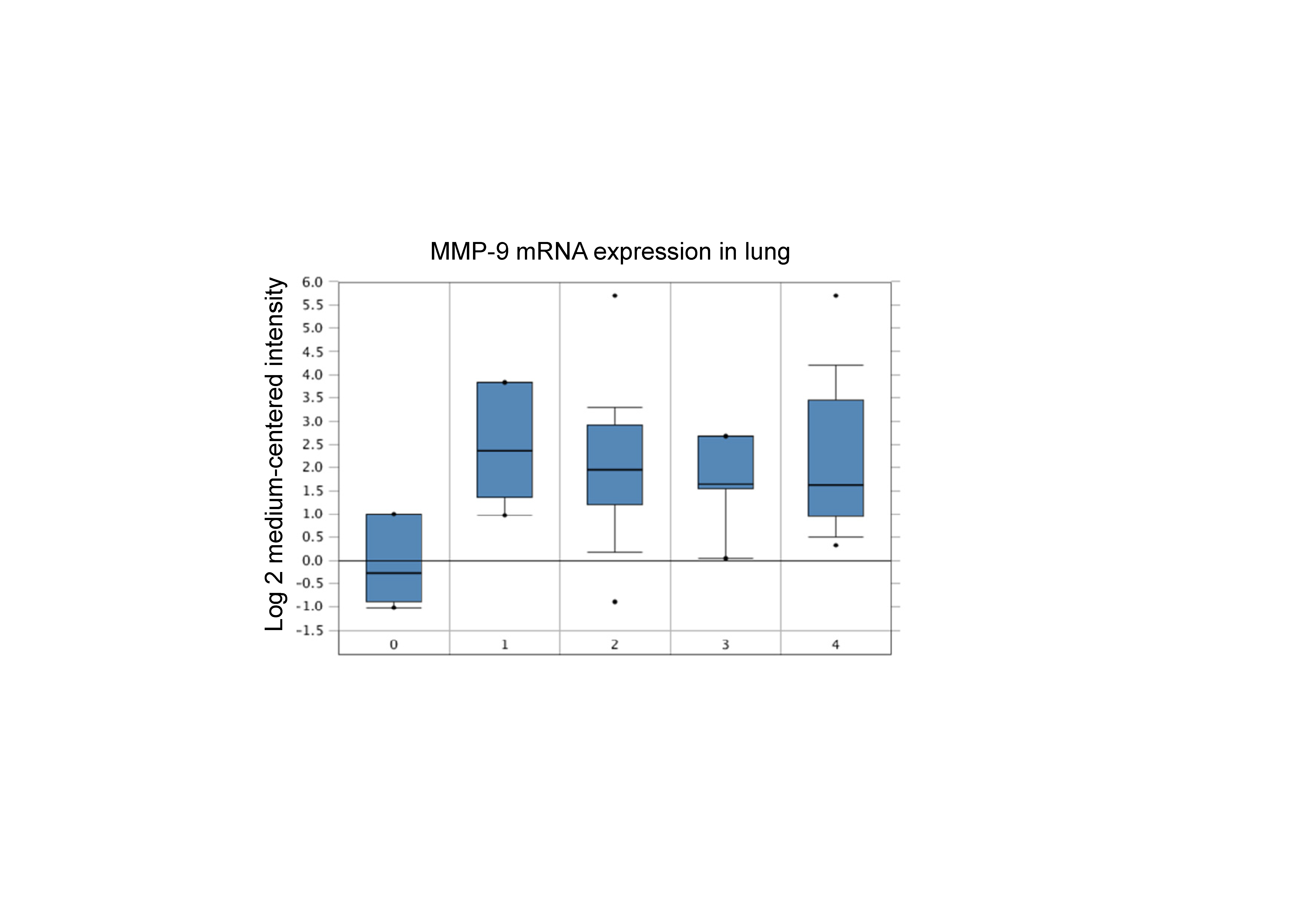

Supplement: S1 Fig — This was a study by Garber et al [16]. Statistical data were retrieved and analyzed from Oncomine, a cancer microarray database and integrated data-mining platform, and results are presented as a box plot diagram. Lung tissues obtained from normal and four lung tumor types were included. 0, normal (n = 6); 1, large-cell lung carcinoma (n = 4); 2, lung adenocarcinoma (n = 42); 3, small cell lung carcinoma (n = 5); 4, squamous cell lung carcinoma (n = 16). The log2 medium-centered intensity means the intensities were processed by median centering (normalization) and then log2-based transformation. (TIF) [file pone.0185021.s001.tif]

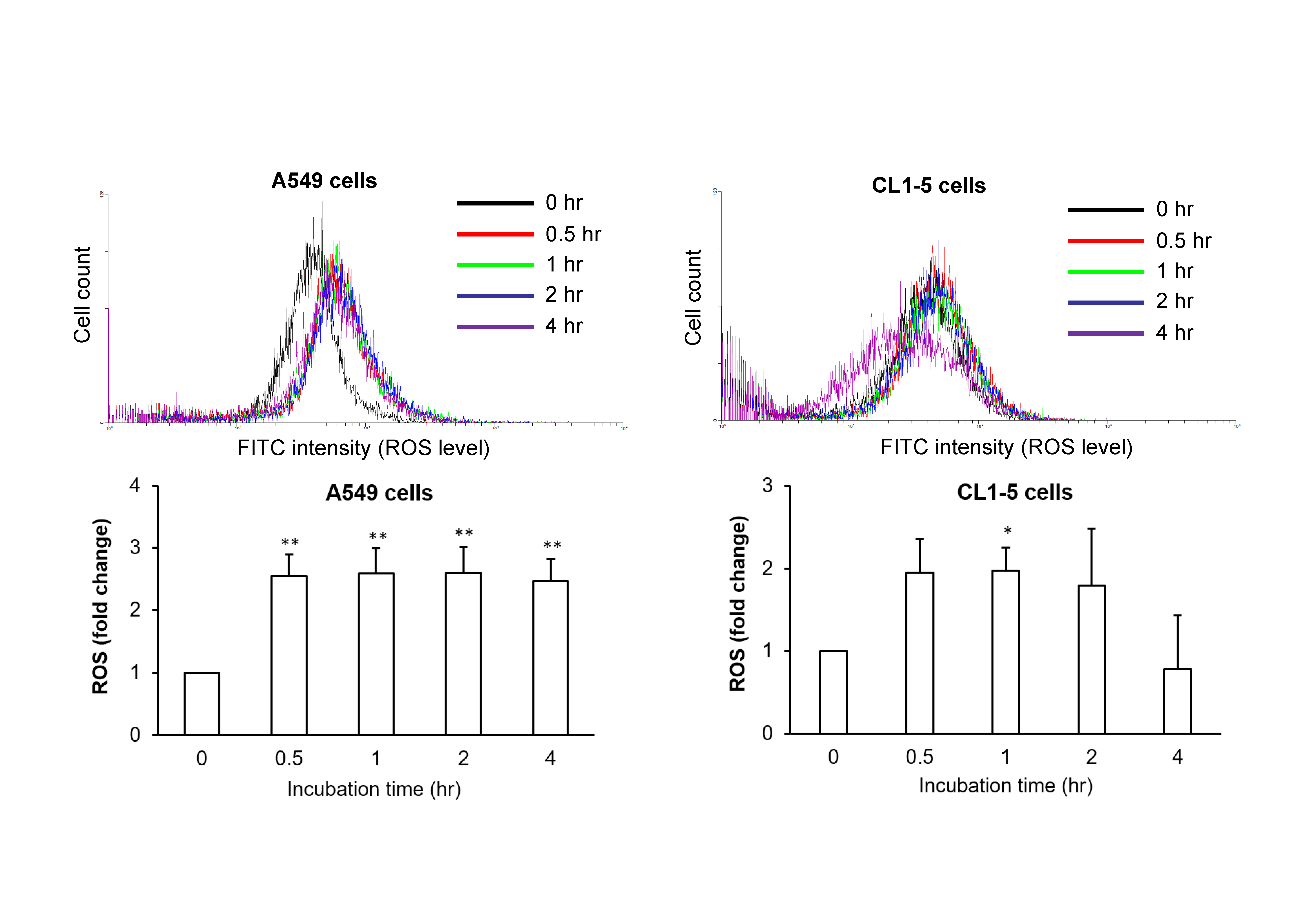

Supplement: S2 Fig — ROS production and changes were measured by flow cytometry in cells treated with 2 μM of NCKU-21 for the indicated period (0~4 h). A detailed description of the measurement of the ROS level is provided in “Supplementary information”. * P < 0.05 and ** P < 0.01, compared to the control group (without NCKU-21 treatment). (TIF) [file pone.0185021.s002.tif]
