# Supplementary material for: Toxicological effects of NCKU-21, a phenanthrene derivative, on cell growth and migration of A549 and CL1-5 human lung adenocarcinoma cells
Source: PLoS One. 2017 Sep 25;12(9):e0185021. doi: 10.1371/journal.pone.0185021 (PMC5612657; doi:10.1371/journal.pone.0185021)
Supplement: S1 File — (PDF) [file pone.0185021.s003.pdf]

Supporting Information For

Toxicological Effects of NCKU-21, a Phenanthrene Derivative, on Cell Growth and Migration of A549 and CL1-5 Human Lung Adenocarcinoma Cells

Short title: Activity of NCKU-21 in lung adenocarcinoma cells

Hsien-Feng Liao 1,2,¶, Chun-Hsu Pan 3,¶, Yi-Fong Chen 1, Tian-Shung Wu 4,5,*, Ming-Jyh Sheu 6,*, Chieh-Hsi Wu 3,*

1 The PhD Program for Cancer Biology and Drug Discovery, China Medical University and Academia Sinica, Taichung, Taiwan

2 Department of Pharmacy, Yuanli Lee’s General Hospital, Lee’s Medical Corporation, Miaoli, Taiwan

3 School of Pharmacy, Taipei Medical University, Taipei, Taiwan

4Department of Chemistry, National Cheng Kung University, Tainan, Taiwan

5 Department of Pharmacy, National Cheng Kung University, Tainan, Taiwan

6 School of Pharmacy, China Medical University, Taichung, Taiwan

¶ Equal contribution as first authors.

***Corresponding Authors:**

E-mail: chhswu@tmu.edu.tw (CHW); E-mail: tswu@mail.ncku.edu.tw (TSW); E-mail: hsumj@mail.cmu.edu.tw (MJS)

Materials and Methods

Measurement of reactive oxygen species (ROS)

An ROS-specific dye, 2′,7′-dihydrofluorescein diacetate (DCFH-DA) (#c363; Life Technologies, Rockville, MD, USA) was used to evaluate the accumulated level of intracellular ROS. Briefly, cells (10^5 cells/well) were treated with 2 μM of NCKU-21 and trypsinized at several time points (0, 0.5, 1, 2, and 4 h post-treatment). Harvested cells were resuspended and incubated in 1 mL of a diluted DCFH-DA solution (20 μM in phosphate-buffered saline (PBS)) at 37 °C for 30 min in the dark. After that, the intracellular fluorescent intensity (accumulation level of ROS) was measured with a flow cytometer.

Results

NCKU-21 induces accumulation of ROS in A549 and CL1-5 cells

Measurement of ROS production was carried out in cells stained with DCFH-DA, an ROS-specific dye, and detected with a flow cytometer. Data showed that the level of ROS had obviously increased by 30 min with 2 μM of NCKU-21 treatment in both cancer cell lines (S2 Fig). At 4 h after treatment, the accumulated level of intracellular ROS was still sustained in A549 cells but had returned to the pretreatment value in CL1-5 cells.
